# Application of An Electrical Resistance Sensor-Based Automated Corrosion Monitor in the Study of Atmospheric Corrosion

**DOI:** 10.3390/ma12071065

**Published:** 2019-04-01

**Authors:** Zhuolin Li, Dongmei Fu, Ying Li, Gaoyuan Wang, Jintao Meng, Dawei Zhang, Zhaohui Yang, Guoqing Ding, Jinbin Zhao

**Affiliations:** 1 School of Automation and Electrical Engineering, University of Science and Technology Beijing, Beijing 100083, China; S20170591@xs.ustb.edu.cn (Z.L.); G20178600@xs.ustb.edu.cn (Y.L.); 841650980@163.com (G.W.); B20160269@xs.ustb.edu.cn (J.M.); 2 Institute for Advanced Materials and Technology, University of Science and Technology Beijing, Beijing 100083, China; dzhang@ustb.edu.cn; 3 Qingdao Marine Corrosion Research Institute, Central Iron and Steel Research Institute, Qingdao 266071, China; yzhaohui1@tom.com (Z.Y.); dinggq99@163.com (G.D.); 4 Research Institute, Nanjing Iron & Steel Co., Ltd., Nanjing 210035, China; zhaojinbin@njsteel.com.cn

**Keywords:** corrosion monitoring, IoT ACM, electrical resistance sensor, steel, atmospheric corrosion, corrosion data

## Abstract

An automated corrosion monitor, named the Internet of Things atmospheric corrosion monitor (IoT ACM) has been developed. IoT ACM is based on electrical resistance sensor and enables accurate and continuous measurement of corrosion data of metallic materials. The objective of this research is to study the characteristics of atmospheric corrosion by analyzing the acquired corrosion data from IoT ACM. Employing data processing and data analysis methods to research the acquired corrosion data of steel, the atmospheric corrosion characteristics implied in the corrosion data can be discovered. Comparing the experiment results with the phenomenon of previous laboratory experiment and conclusions of previously published reports, the research results are tested and verified. The experiment results show that the change regulation of atmospheric corrosion data in the actual environment is reasonable and normal. The variation of corrosion depth is obviously influenced by relative humidity, temperature and part of air pollutants. It can be concluded that IoT ACM can be well applied to the conditions of atmospheric corrosion monitoring of metallic materials and the study of atmospheric corrosion by applying IoT ACM is effective and instructive under an actual atmospheric environment.

## 1. Introduction

Real-time and on-line corrosion monitoring are some of the focuses of scientific research in informatics for materials corrosion and protection [1]. Acquiring accurate corrosion data in an actual atmospheric environment rather than a laboratory environment is the priority and difficulty in the corrosion monitoring engineering. Researchers can analyze corrosion data acquired by appropriate technology and device to discover corrosion characteristics [2,3]. In off-line monitoring techniques the time interval of weight measurement and thickness loss measurement is long, so off-line monitoring techniques are incapable of revealing unforeseeable changes which could develop during corrosion progress [4,5]. On-line monitoring techniques, such as electrochemical impedance measurement, electrochemical frequency modulation, polarization resistance measurement, microwave imaging technology, photoacoustic sensor, fiber grating sensor and fiber optic sensor [6,7,8,9,10,11,12] are all delicate and expensive because they require auxiliary experimental equipment and special experimental conditions. The above-mentioned techniques can obtain excellent measurement results in a laboratory environment, but in an actual monitoring environment the measurement results would be not satisfactory. Electrical resistance sensor is another on-line monitoring technique which provides a simple and efficient measurement method for real-time corrosion monitoring [13,14,15]. With the rapid development of design philosophy and technology, state-of-the-art electrical resistance sensor can not only accurately acquire corrosion data, but also adapt to a variety of corrosion monitoring conditions, including the actual atmospheric environment.

Nowadays, some mature atmospheric corrosion monitor (ACM) products based on electrical resistance sensor have been developed and the most representative ones are AirCorr and GR6000 [16,17,18,19]. However, there have been no reports of these products being used in a real and severe atmospheric environment and the above products have some defect, such as restricted measurement conditions and poor degree of automatic and information. Besides, in a harsh outdoor environment, atmospheric corrosion of metallic materials will be severe, it is inconvenient for researchers to continuously carry out corrosion monitoring work in this situation at a long time. Considering all the above problems, our research team has invented a new device based on a state-of-the-art electrical resistance sensor, which adopts a new design concept and integrates the advantages of advanced sensing technology, intelligent control technology and IoT technology. So IoT ACM has the ability to accurately and continuously acquire massive corrosion data and transmit them through the Internet in real time. Our purpose of developing IoT ACM is to make up for the shortcomings in existing products of the same type. IoT ACM can break through the limitation of time and space for atmospheric corrosion monitoring progress and IoT ACM is able to provide an automatic and professional measurement approach.

In this study, characteristics of atmospheric corrosion of Q235 steel under actual atmospheric environment was analyzed by researching acquired corrosion data from IoT ACM. Firstly, denoising corrosion data to remove the high frequency noise and then study the change regulation to analyze some common phenomena of corrosion data in the case of using IoT ACM in an actual atmospheric environment. This experiment can provide new and effective corrosion data processing and research method and can prove the change regulation of corrosion data is reasonable and normal. Secondly, researching the correlational relationship between corrosion depth and atmospheric environmental elements data by visualization analysis and data mining methods and then discover the implied relationship between atmospheric corrosion of steel and the atmospheric environment. Through the analysis of the experimental results, the extent of influence of atmospheric environmental elements on atmospheric corrosion of steel can be obtained.

## 2. Experimental Principle and Details

### 2.1. Experimental Overview

**Figure 1:**
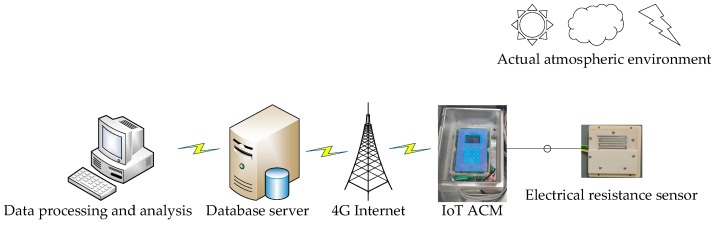
The schematic illustration of performance analysis experiment. IoT ACM, Internet of Things atmospheric corrosion monitor.

In this study, research of atmospheric corrosion by IoT ACM is planned to be carried out in an actual atmospheric environment. IoT ACM will acquire the atmospheric corrosion data of Q235 steel by electrical resistance sensor. Then the corrosion data will be transmitted to a customized database server through the Internet and the data will be used to discover atmospheric corrosion characteristics of Q235 steel by data processing and data analysis methods. The schematic illustration of this experiment is shown in Figure 1.

### 2.2. Electrical Resistance Sensor

**Figure 2:**
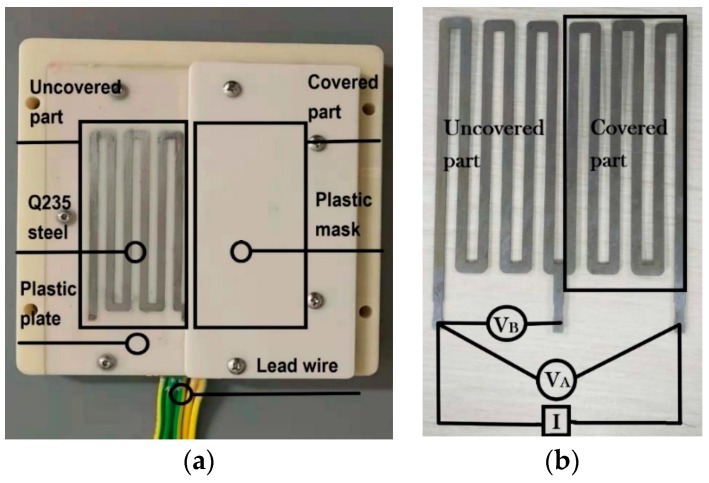
The schematic illustration of an electrical resistance sensor. (**a**) The appearance of an electrical resistance sensor; (**b**) Schematic diagram of detection principle of electrical resistance sensor.

Electrical resistance sensor is an important component of IoT ACM. This sensor is a kind of state-of-the-art patented product, which can eliminate the influence of temperature change, obtain higher measurement accuracy and ensure uniform corrosion as much as possible. According to the ASTM standard B 829-09(2015) [20], the electrical resistance sensor is made of a comb shaped Q235 steel that is inlaid in a plastic plate. Selecting an appropriate initial dimension of Q235 steel, the electrical resistance sensor can be applicable in various monitoring conditions and the service life of electrical resistance sensor means the time to corrode a half of the thickness of the Q235 steel. In this sensor one part of the steel is covered by a plastic mask which provides a good seal to prevent steel from directly attacking in atmospheric conditions. In the monitoring progress, the covered part performs as a reference for evaluating changes in the uncovered part. The ratio of voltage (($V_{A}$/$V_{B}$), hereinafter, corrosion data) about the series voltage of covered part and uncovered part (expressed as $V_{A}$) to the voltage of the uncovered part (expressed as $V_{B}$) provides a measurement of the amount of Q235 steel that has reacted with atmospheric environment, which provides a measurement of atmospheric corrosion [13,14,17]. In the actual measurement process, the electrical resistance sensor is powered by a constant current source, so the resistance of Q235 steel is proportional to the resistance voltage. And taking advantage of the form of voltage ratio, the corrosion data acquired by an electrical resistance sensor can eliminate the influence caused by the change of temperature and have a function of temperature compensation. Figure 2 shows a schematic illustration of the electrical resistance sensor.

The purpose of measurement on corrosion data is to calculate the corrosion depth Δ*h* of Q235 steel and Δ*h* can be expressed according to the following formula

(1) $$\begin{array}{c} \Delta h\;=\;(1-\frac{K_{0}}{K_{t}}{)h}_{0}\;\\ k_{0}\;=\;\frac{V_{B0}}{V_{A0}{-V}_{B0}}\;=\;\frac{R_{\mathrm{corr},\mathrm{init}}}{R_{\mathrm{ref},\mathrm{init}}}\\ k_{t}\;=\;\frac{V_{\mathrm{Bt}}}{V_{\mathrm{At}}{-V}_{\mathrm{Bt}}}\;=\;\frac{R_{\mathrm{corr},t}}{R_{\mathrm{ref},t}} \end{array}$$

where $h_{0}$ is the initial thickness of Q235 steel at the beginning of measurement, $K_{0}$ is the value of initial voltage ratio and it is a constant value, $K_{t}$ is the value of current voltage ratio. $V_{A0}$ is the initial measured series voltage of uncovered part and covered part, $V_{B0}$ is the initial measured voltage of the uncovered part, $R_{\mathrm{corr},\mathrm{init}}$ is the initial resistance of the uncovered part, $R_{\mathrm{ref},\mathrm{init}}$ is the initial resistance of the covered part. $V_{\mathrm{At}}$ is the current measured series voltage of uncovered part and covered part, $V_{\mathrm{Bt}}$ is the current measured voltage of the uncovered part, $R_{\mathrm{corr},t}$ is the current resistance of the uncovered part and $R_{\mathrm{ref},t}$ is the current resistance of the covered part.

There is a physical phenomenon that the resistance of Q235 steel will be affected by temperature and the formula of resistance changing with temperature is shown in Equation (2)

(2) $$R_{t}\;=\;\left(1\;+\;\alpha T\right)R_{0}\;$$

where $R_{0}$ is the initial resistance of Q235 steel, $R_{t}$ is the current resistance of Q235 steel, T is the temperature and α is the temperature coefficient of resistance of steel. Besides, the covered part and the uncovered part are all in the same temperature condition, so the form of voltage ratio in the measurement progress can play an important role in temperature compensation to eliminate the influence caused by the change of temperature. According to the principle of temperature compensation, the Equation (1) can be expressed by the following equation,

(3) $$\Delta h\;=\;(1\;-\frac{R_{\mathrm{corr},\mathrm{init}}}{R_{\mathrm{ref},\mathrm{init}}}\text{·}\frac{R_{\mathrm{ref},\mathrm{init}}}{R_{\mathrm{corr}}}{)h}_{0}\;=\;(1-\frac{R_{\mathrm{corr},\mathrm{init}}}{R_{\mathrm{corr}}})$$

### 2.3. IoT ACM

**Figure 3:**
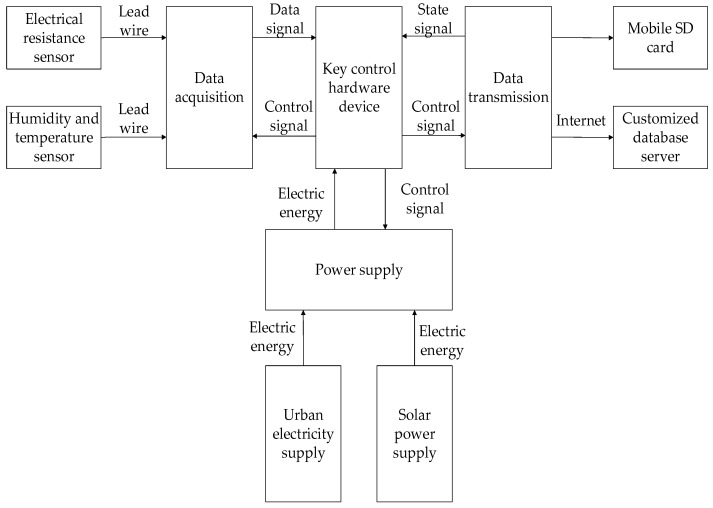
The block diagram of IoT ACM.

IoT ACM is the latest patented product and it consists of a key controlling hardware device, a data acquisition system, a data transmission system and a power supply system. Figure 3 shows the block diagram of IoT ACM. The core component of IoT ACM is the key controlling hardware device which integrates the functions, including core control, data acquisition, data transmission, power supply and so on. And the hardware device has the ability to complete the established functions of IoT ACM, which are reflected in real time, high precision and high efficiency. For the purpose of protecting hardware device in IoT ACM, an industrial-grade double layer protective shell is designed, of which the outer shell is made of aluminum alloy and the inner one is made of plastic. So the protective shell can prevent the hardware from external impact, moisture and dust in the environment. The corrosion data will be acquired by an electrical resistance sensor via the lead wire connected with the key controlling hardware device and recorded in a logger at an interval of one hour. Besides, for the comprehensiveness of science research, a humidity and temperature sensor is also installed in IoT ACM and the temperature (hereinafter, T) data and relative humidity (hereinafter, RH) data will also be acquired using the same method. There are two ways to export the data collected in the logger of IoT ACM. The first one is using the mobile mass storage SD card implanted in IoT ACM. And the second one is embodying the design concept of IoT ACM. Using a 4G network communication module, IoT ACM can transmit data stably and quickly via a 4G mobile network, especially for the simultaneous transmission of massive data. Through the Internet, corrosion data will be shorted in a customized database server to support sharing corrosion data from a scientific research data management platform [21]. The power supply system consists of an industrial-grade power supply module using the urban electricity supply and an auxiliary solar power supply battery. Therefore, the power supply system ensures the all-weather uninterrupted operation of IoT ACM in the monitoring progress at any field test sites [22]. 

### 2.4. Field Test Details

**Figure 4:**
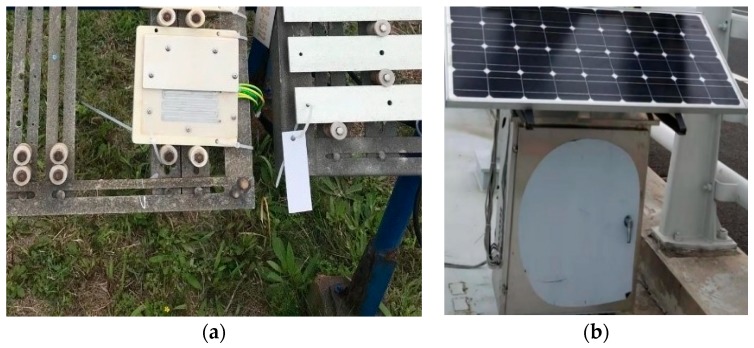
The field test details. (**a**) Electrical resistance sensor before experiment; (**b**) Appearance of IoT ACM.

An IoT ACM was placed in Qingdao, eastern China [23]. The continuous monitoring of atmospheric corrosion of Q235 steel went on for more than three months in the vicinity of the fourth quarter in 2017 (from September 24th to December 31st). Electrical resistance sensor was installed in a professional exposure frame by some dielectric clips, which faced the sun and inclined at a 45-degree angle to the horizontal. IoT ACM was placed approximately 10 meters near the exposure frame. IoT ACM and electrical resistance sensor were connected by industrially advanced lead wires to ensure the validity of transmission of acquired corrosion data. Figure 4 shows the field test details. In this study, the dimensions about Q235 steel of electrical resistance sensor were 70 mm × 70 mm × 0.15 mm (length × width × height), for which the electrical resistance sensor had a high origin resistance that could resist the influence of circuit in IoT ACM during the measurement progress and had the ability to achieve uniform corrosion and avoid the generation about local corrosion as much as possible. Besides, the dimensions of the plastic mask were 129 mm×129 mm×14 mm and the dimensions of the plastic mask were 129 mm × 65 mm × 5 mm. The plastic plate and plastic mask were fastened by some screws and the gaps between them were sealed with a silicone seal to prevent the covered part of Q235 steel in the sensor from the atmospheric corrosion conditions.

In Qingdao the air pollution also might have a serious impact on the atmospheric corrosion of steel [24,25,26]. Therefore, in this research, collecting contaminants (PM2.5, PM10, SO_2_, NO_2_) data of air pollution from Qingdao Laoshan District Substation of the National Urban Air Quality Real-time Publishing Platform during the progress of atmospheric corrosion monitoring experiment to discover the correlation between corrosion of Q235 steel and the air pollution [27]. The distance between air pollution measurement unit and field test site is 5.4 km. The field test site is within the monitoring range of the air pollution measurement unit in the substation, so the collected contaminants (PM2.5, PM10, SO_2_, NO_2_) data of air pollution can be used in this study.

## 3. Results and Discussions

### 3.1. Study on the Change Regulation of Atmospheric Corrosion Data

**Figure 5:**
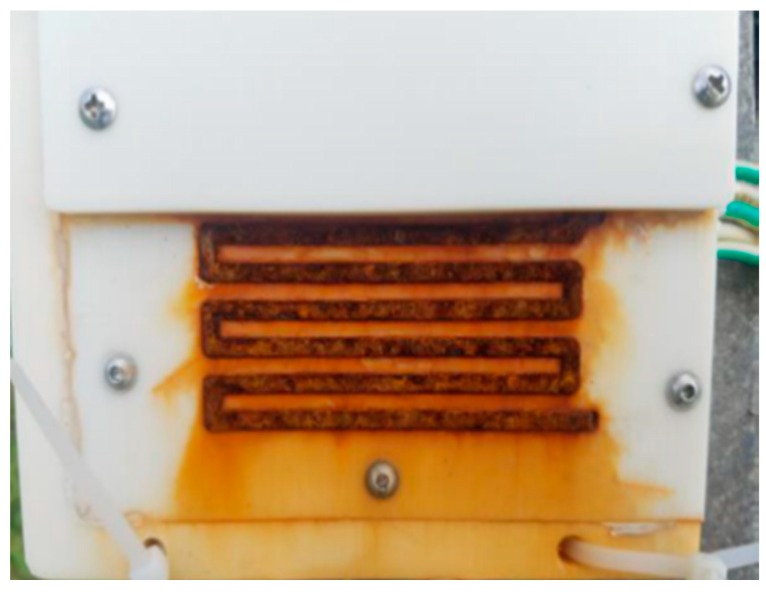
Picture of electrical resistance sensors after the experiment (exposed 99 days).

The change regulation of corrosion data could imply some characteristics of atmospheric corrosion. The characteristics of atmospheric corrosion of metallic materials can be obtained by analyzing the variation of corrosion data. According to Equations (1) and (3), in the corrosion progress the thickness of the uncovered part decreases and the resistance of it increases, which could result in a continuous decrease in the value of corrosion data. The appearance of an electrical resistance sensor of IoT ACM after the experiment under actual atmospheric environment is shown in Figure 5. A large amount of red rust is observed on the uncovered part in the sensor. This phenomenon can indicate the change trend presented by the corrosion data is normal. In Figure 5, it is obvious that the plastic mask is intact and there is no lifting, misalignment or damage and it can be proved that the corrosion data acquired by IoT ACM is reliable and effective. However, the corrosion data collected by the IoT ACM in the actual environment still have quasi-periodic pulses which are similar to the phenomenon in the previous laboratory environment. In the laboratory environment, Q235 steel is only affected by temperature, so quasi-periodic pulses in corrosion data are considered to be caused by the change of temperature in one day [28]. In an actual atmospheric environment, atmospheric environmental elements (RH, T and air pollutants) may also have an impact on the cause of quasi-periodic pulses, so this section is to research characteristics of acquired data to verify the change regulation of atmospheric corrosion data is correct and normal. 

**Figure 6:**
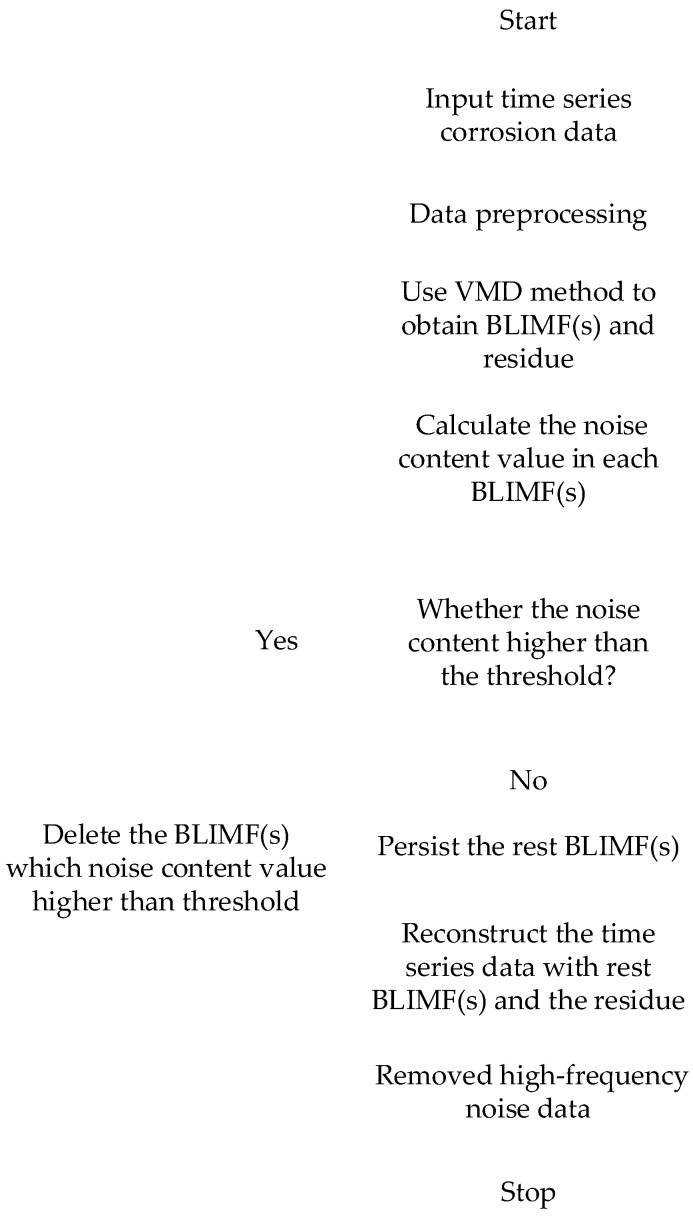
Flowchart of an adaptive denoised method based on variational mode decomposition (VMD).

Data smoothing method is often used in practical engineering application to deal with corrosion data acquired by electrical resistance sensor [29], but it could omit some detailed information, such as the pulses appearing as a quasi-periodic form. Taking into account the characteristics of corrosion data, the high frequency noise caused by electronic components must be removed firstly, so that the quasi-periodic pulses could clearly emerge. In order to accurately remove the interference of high frequency noise and prevent important information from destructing, the common low pass filter is not selected in this study. Therefore, an adaptive data denoised method based on variational mode decomposition (VMD) is used in this research, Figure 6 shows the flowchart of this method. VMD is a modern time series data (herein, corrosion data essentially belongs to time series data) decompose technique and has complete mathematical theory, explicit physical meaning and good practicality. The decomposition process is essentially a special iteration solving variational mode and can non-cursively decompose a multicomponent time series data into series of band-limited intrinsic mode functions (BLIMFs). In order to establish a variational model of time series data decomposition, the VMD defines the BLIMF as the amplitude modulation-frequency modulation (AM-FM) and the core principles are expressed as follows:

(4) $$\begin{array}{c} u_{k}\left(t\right){\;=\;A}_{k}\left(t\right){\cos(\varphi }_{k}\left(t)\right)\\ \omega_{k}\left(t\right){\;=\;d\varphi }_{k}\left(t\right)/dt\;\geq \;0\\ \underset{\left\{u_{k}\right\},\left\{\omega_{k}\right\}}{\min}\left\{\overset{K}{\underset{k=1}{\sum }}\|\partial_{t}{\left[\left(\delta \left(t\right)+\frac{j}{\pi t}\right){*u}_{k}\left(t\right)e^{{-j\omega }_{k}t}]\right\|}_{2}^{2}\right\}\\ \overset{K}{\underset{k=1}{\sum }}u_{k}\left(t\right)\;=\;f(t)\\ x(t)\;=\;f(t)\;+\;\lambda (t) \end{array}$$

where $A_{k}\left(t\right)$ is the instantaneous amplitude of $u_{k}\left(t\right)$ and $\varphi_{k}\left(t\right)$ is the instantaneous phase of $u_{k}\left(t\right)$, $\varphi_{k}\left(t\right)$ is the reduction function that instantaneous frequency $\omega_{k}\left(t\right){=d\varphi }_{k}\left(t\right)/\mathrm{dt}\;\geq \;0.$ On the basis of this definition, the VMD assumes that the input time series data x(t) is composed of finite BLIMF components with different center frequencies and limited bandwidths and the minimum sum of the estimated bandwidth of each BLIMF component is almost equal to the input time series data x(t). K is the number of BLIMF component, $\left\{u_{k}\right\}=\left\{u_{1}{,u}_{2}{,\text{…},\;u}_{K}\right\}$ are BLIMF components and $\left\{\omega_{k}\right\}=\left\{\omega_{1}{,\omega }_{2}{,\text{…},\;\omega }_{K}\right\}$ are center frequencies of $u_{k}\left(t\right)$. δ(t) is Dirichlet function, ∗ symbol is convolution symbol and λ(t) is the residue which has less information of x(t) [30].

**Figure 7:**
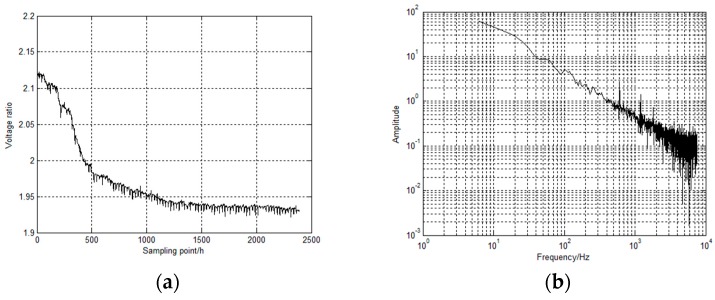

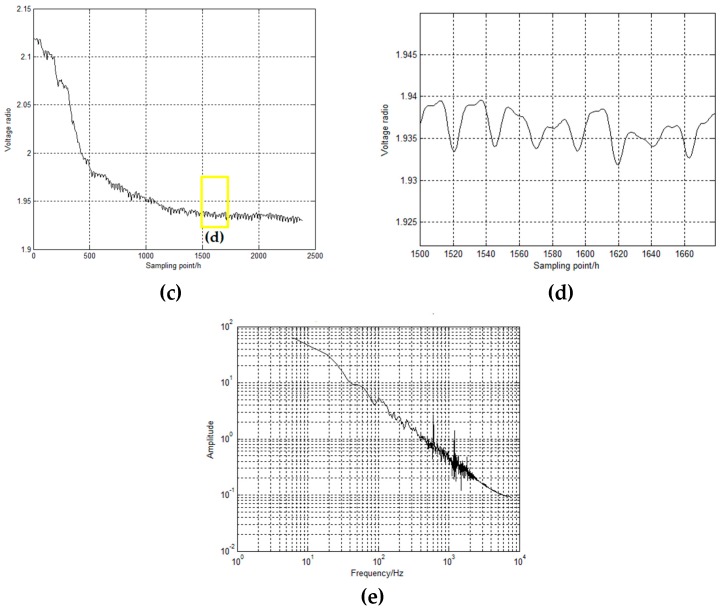
The denoised corrosion data. (**a**) Origin corrosion data; (**b**) Origin corrosion data spectrum(log-log); (**c**) Denoised corrosion data; (**d**) Detailed denoised corrosion data. (**e**) Denoised corrosion data spectrum (log-log).

The processing result of the VMD method will be not ideal for denoising low signal to noise ratio (SNR) data and the outliers could have a devastating effect on the decomposition result. Therefore, it is important to remove the background noise and outlier interference in time series data using the preprocessing method [31]. For the purpose of getting the optimal data processing effect, the VMD method decomposes time series data after preprocessing into 10 number of BLIMFs, for which it is mostly suitable for acquired corrosion data. From BLIMF1 to BLIMF10, the center frequencies of them gradually increase, the BLIMF1 is the basis-frequency BLIMF. A mathematical index described as long-range correlation provides an effective way to quantify the noise containing condition in each BLIMF. Comparing the calculation results with a specified threshold, it is possible to judge whether a BLIMF is seriously influenced by noise interference or not. The value of the threshold is usually set to 0.5, if the value of mathematical index < 0.5, it indicates the BLIMF contains more noise interference, if the value≧0.5, it indicates that the BLIMF can be considered as no noise interference vice versa [32]. The calculation results show that BLIMF1, BLIMF2 and BLIMF3 have such excellent quality. Reconstructing them with the residue can obtain removed high-frequency noise corrosion data and the detailed results are shown in Figure 7.

Comparing with Figure 7b,e, the high-frequency noise has been removed. But in Figure 7c, there are obvious quasi-periodic pulses in the denoised corrosion data and the detailed information about the period of each pulse, shown in Figure 7d, is almost close to 24*h*. In the actual environment, the variation of atmospheric environmental elements will exhibit a certain periodicity, such as the cycle of days, weeks and months. So it is very significant to analyze the impact of cyclical atmospheric environmental elements on the corrosion data. Selecting a piece of denoised corrosion data which has no obvious inherent trend of the corrosion data (the denoised corrosion data is selected from sampling point 1500 to the end). In this paper, the two-dimension correlation coefficient (TDC) is used to analyze the relationship between corrosion data and atmospheric environmental data and investigate which atmospheric environmental elements for a period of days, weeks and months may cause quasi-periodic pulses in the denoised corrosion data [33]. For computational requirements of TDC method, splitting the experimental data (herein, denoised corrosion data and atmospheric environmental elements data are all selected from sampling point 1500 to the end) in days (weeks or months) and create *m* × *n* matrix, of which *m* represents how many days (weeks or months) and *n* represents how many hours in one day (one week or one month). The calculation principle of TDC is as follow:

(5) $$\begin{array}{c} r_{h}\;=\;\frac{\sum_{m}\sum_{n}\left(A_{\mathrm{mn}}-\bar{A_{m}}\right)\left(B_{mn}-\bar{B_{m}}\right)}{\sqrt{\left[\sum_{m}\sum_{n}{\left(A_{mn}-\bar{A_{m}}\right)}^{2}\right]}\sqrt{\left[\sum_{m}\sum_{n}{\left(B_{mn}-\bar{B_{m}}\right)}^{2}\right]}}\\ r_{v}\;=\;\frac{\sum_{m}\sum_{n}\left(A_{\mathrm{mn}}-\bar{A_{n}}\right)\left(B_{mn}-\bar{B_{n}}\right)}{\sqrt{\left[\sum_{m}\sum_{n}{\left(A_{\mathrm{mn}}-\bar{A_{n}}\right)}^{2}\right]}\sqrt{\left[\sum_{m}\sum_{n}{\left(B_{mn}-\bar{B_{n}}\right)}^{2}\right]}} \end{array}$$

where $r_{h}$ is the horizontal correlation and $r_{v}$ is the vertical correlation between matrix ***A*** and ***B*** which have the same size. The $\bar{A_{m}}$ is the average of *m*th row of matrix ***A*** and the $\bar{A_{n}}$ is the average of *n*th column of matrix ***A***. The $\bar{B_{m}}$ is the average of *m*th row of matrix ***B*** and the $\bar{B_{n}}$ is the average of *n*th column of matrix ***B***. The values of $r_{h}$ and $r_{v}$ can take values from −1 to +1. The greater the absolute values of $r_{h}$ and $r_{v}$ are greater the correlation can be reflected [25]. In the results of TDC, the following symbols are used to analyze the relationship between corrosion data and atmospheric environmental elements in different time units (day, week and month). For example, the change of one of the atmospheric environmental elements in one day can be expressed as *X*_data_, in one week can be expressed as *X*_week_ and in one month can be expressed as *X*_month_, where X can represent T, RH, PM2.5, PM10, SO_2_ and NO_2_ in this paper. 

In this paper, the value of $r_{h}$ index indicates whether the atmospheric environmental elements can cause the occurrence of quasi-period pulses or not, the value of $r_{v}$ index reveals what atmospheric environmental elements influence the amplitude of pulses in the corrosion data (except for the condition of monthly). The results of TDC analysis for a of days, shown in Table 1, the absolute value of $r_{h}$ and $r_{v}$ about T_day_ are evidently higher than the calculating result of other atmospheric environmental elements. The results of TDC analysis for a period of weeks, shown in Table 2, the absolute value of $r_{h}$ about T_week_ and $r_{v}$ about RH_week_ are evidently higher than the calculating result of other atmospheric environmental elements. According to the comprehensive analysis of the above experimental results, in Table 1, it can be concluded that the quasi-periodic pulses are most likely caused by T_day_ in the atmospheric environment and the experimental result is consistent with results of IoT ACM in previous laboratory experiment and the transmission delay of temperature on the steel of sensor could generate quasi-periodic pulses. In Table 2, when atmospheric environment elements change for a period as weeks, T_week_ may cause fluctuations in corrosion data, but such fluctuations are reflected in the average T during this period. In general, an increase in T within a certain range will accelerate atmospheric corrosion of carbon steel and vice versa. In addition, the RH_week_ has a great influence on the corrosion rate in the initial stage of atmospheric corrosion of carbon steel. In Table 3, when atmospheric environment elements change for a period as months (due to the amount of data, only one month’s data was used in this experiment, so $r_{v}$ cannot be calculated), similar to the results in Table 2, the overall changes in RH_month_ and T_month_ may have an impact on the corrosion data. However, changes on a weekly or monthly basis will affect the overall corrosion rate more and will not affect the cause of quasi-periodic pulse of corrosion data, which the period of them is almost closed to 24*h.*

It can be known from the above analysis that the change regulation of the corrosion data conforms to common sense, so the corrosion data collected under the actual environment is effective and normal and can be used for scientific research.

### 3.2. Study on the Influence of Atmospheric Environmental Elements on Corrosion

In this paper, this part of the experimental results is used as an example application of continuous monitoring by IoT ACM. Atmospheric environmental elements have the ability to influence atmospheric corrosion of steel and atmospheric corrosion characteristics can be obtained by studying the relationship between corrosion data and environmental data. By analyzing the relationship between atmospheric environmental elements and corrosion data in this special experimental environment and time, it is possible to find out the characteristics of atmospheric corrosion of steel under a certain circumstance. Data visualization analysis is an intuitive method to study the correlation between corrosion data and atmospheric environmental elements. Using data visualization method can discover the relationship between atmospheric environmental elements and processed corrosion data which is smoothed by standard smoothing algorithm ‘RLOESS’ provided by MATLAB R2014a to smooth the quasi-periodic pulses.

In the atmospheric corrosion progress of steel, RH is one of the major influence factors [13,17,34]. RH has a critical point, the atmospheric corrosion will happen when the value of RH is higher than the critical point. Generally speaking, RH critical point of metallic materials is in a range between 50% to 70% and the critical point of steel is 65% in particular, but when encountering some case of serious air pollution, the value of critical point will decrease [35]. Atmospheric corrosion of steel is progress evidently reflected by the change of RH and it tends to exhibit a higher corrosion rate under higher RH conditions in the initial stage [36].

**Figure 8:**
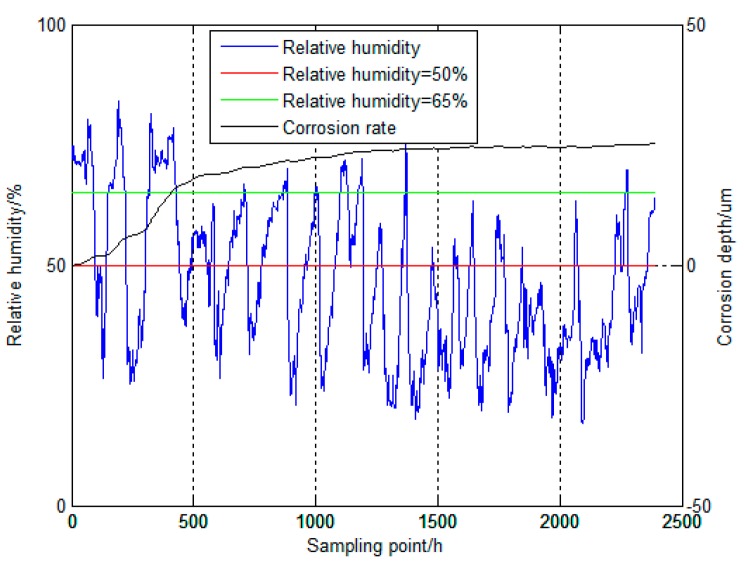
Detailed information about corrosion depth and RH.

The processed corrosion data can be converted into the form of corrosion depth and the corrosion depth has a rapidly increase phase and mildly increase phase. According to the analysis of Figure 8, it can be concluded that, when RH value is high, the corrosion depth changes dramatically, when the RH value is low, the corrosion depth hardly changes substantially. By comparing it with existing knowledge, in the initial stage of atmospheric corrosion of steel, when RH value is higher than 65% and it lasts in a long time, the corrosion depth will be in the rapidly increase phase and the corrosion rate will accelerate and reach the crest value. Besides, when RH value is lower than 65%, but higher than 50%, the corrosion depth will begin to mildly increase and when the RH value is lower than 50%, the corrosion depth will hardly increase.

**Figure 9:**
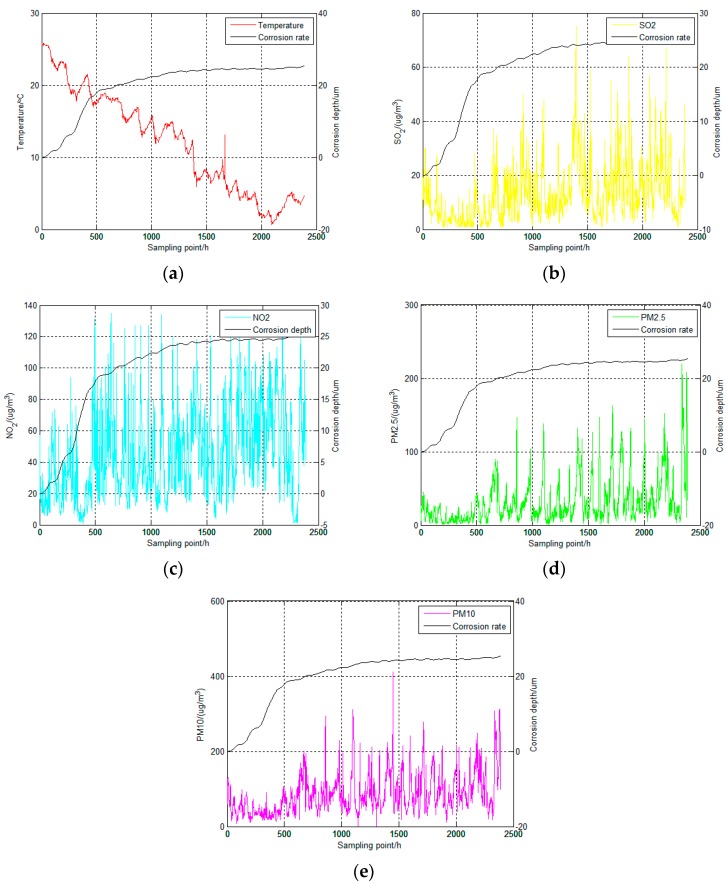
Detailed information about corrosion depth and atmospheric environmental elements. (**a**) The relationship between corrosion depth and T; (**b**) The relationship between corrosion depth and SO_2_; (**c**) The relationship between corrosion depth and NO_2_; (**d**) The relationship between corrosion depth and PM2.5; (**e**) The relationship between corrosion depth and PM10.

In addition to RH, other atmospheric environmental elements are used data visualization method to analyze the relationship between them and atmospheric corrosion. According to Figure 9a, it is evident that T and corrosion depth have a strong correlation and this analysis result is similar to the public awareness [24]. But in Figure 9b–e, the corrosive gas (SO_2_, NO_2_) and particulate matters (PM2.5, PM10) show no obvious changes and perform as a form of white noise. So the visualization analysis results about the corrosive gas and particulate matters have an unobvious relationship with corrosion depth and the existing research results have no ability to provide a clear assessment about the visualization result [37,38,39]. Therefore, it is possible that the relationship between these elements and corrosion depth cannot be obtained by simple visualization analysis.

In order to carry out deeper research, atmospheric environment elements are used to establish correlation models with corrosion depth to discover the implied information that is unable to observed by visualization analysis. Maximal information coefficient (MIC) is a modern correlation analysis method and it can measure all linear and nonlinear relationship between two variables [40]. The principle of MIC is expressed as Equation (6)

(6) $$\begin{array}{c} {M(A,B|D)}_{i,j}\;=\;\frac{I^{*}\left(A,B,D,i,j\right)}{logmin(i,j)}\\ MIC(A,B|D)\;=\;\underset{i\times j<B(n)}{max}{\left\{M(A,B|D\right)}_{i,j}\} \end{array}$$

where gives a finite set D of ordered pairs about two variables A and B, parting the *a*-value of D into *a* bins and the *b*-value of D into *b* bins, which allows empty bins. Such a pair of partitions is called an *a*-by-*b* grid *G*. ${I(A,B,D|}_{G},i,j)$ denotes the mutual information of random variables *A* and *B* over grids *G* with *i* rows and *j* columns. The characteristic matrix M(D) of a set *D* of two variables *A* and *B* is an infinite matrix with entries (i,j) and $I^{*}\left(A,B,D,i,j\right)=maxI\left({A,B,D|}_{G},i,j\right)$ is the maximum mutual information of random variables *A* and *B* over all grids *G* with *i* rows and *j* columns. At the same time, the calculated value of MIC is between 0 and 1, 0 means completely independent and 1 means completely dependent or not independent [41]. A correlation analysis experiment is designed using corrosion depth and atmospheric environmental elements. In this experiment, MIC is used to analyze the implied information about all corrosion depth (Part 1), part of the rapidly increasing phase of corrosion depth (Part 2) and part of the mildly increasing phase of corrosion depth (Part 3), respectively. Table 4 shows the results of this experiment.

Through comprehensive analysis of the experiment results, it can be concluded that in the progress of application of continuous monitoring by IoT ACM, MIC could reflect the characteristics of atmospheric corrosion of steel in the initial stage, especially the influence of RH and T. In other words, among all the atmospheric environmental elements, RH and T play a leading role. Besides, the atmospheric pollutants, including SO_2_, NO_2_, PM2.5 and PM10 all have impacts on atmospheric corrosion of Q235 steel and the MIC analysis results are similar, which means that they may have same important to contribution for corrosion progress. SO_2_ is an important atmospheric corrosive gas. Under the same conditions, the influence of SO_2_ on atmospheric corrosion of steel is far more than that of NO_2_ [42]. However, in the application of continuous monitoring with IoT ACM, under the special urban environment and time conditions, the content of SO_2_ in the atmosphere is lower than that of NO_2_ and the corrosion effect under low SO_2_ content may be closed to that under high NO_2_ content [38,42]. In addition, the corrosion effect of SO_2_ and NO_2_ may be enhanced under the condition that they are mixed in an actual atmospheric environment [43]. PM2.5 and PM10 are the main components of haze which are a serious environmental problem. As particulate matters, they are easy to settle to the surface of steel [38]. PM2.5 and PM10 can increase the surface humidity through moisture absorption, promote the dissolution of soluble salts in particulate matter, release corrosive ions and so on to promote the atmospheric corrosion of carbon steel [39]. Therefore, in this monitoring progress, the corrosion contribution of PM2.5, PM10, SO_2_ and NO_2_ may be the same.

## 4. Conclusion 

The atmospheric corrosion of Q235 steel is analyzed by using IoT ACM under an outdoor actual atmospheric environment. The remarkable findings obtained in this study are summarized as follows.

- Corrosion data acquired by IoT ACM is effective and normal, which can be used for researching atmospheric corrosion of metallic materials after proper processing methods.
- The results about the study of atmospheric corrosion of metallic materials using IoT ACM are consistent with the phenomenon of previous laboratory experiment and conclusions of previously published reports. Using corrosion data can quantify the extent to which atmospheric environmental elements affect the atmospheric corrosion of metallic materials in the initial stage under an actual atmospheric environment.
- IoT ACM can realize real-time and on-line remote monitoring of corrosion data in any atmospheric environment and can replace the metallic material of the electrical resistance sensor to measure atmospheric corrosion data of different metals. Using IoT ACM can provide a new approach for corrosion monitoring, accelerate the progress of scientific research and anticorrosive work and save experiment and engineering cost.

## Figures and Tables

**Table 1:** The result of two-dimension correlation coefficient (TDC) analysis (day).

	T_day_	RH_day_	PM2.5_day_	PM10_day_	SO_2,day_	NO_2,day_
$r_{h}$	−0.262	−0.044	0.092	0.041	0.027	0.028
$r_{v}$	0.846	0.336	−0.147	−0.122	−0.274	−0.133

**Table 2:** The result of TDC analysis (week).

	T_week_	RH_week_	PM2.5_week_	PM10_week_	SO_2,week_	NO_2,week_
$r_{h}$	−0.314	−0.252	0.116	0.110	0.030	0.115
$r_{v}$	0.165	0.372	0.016	0.021	−0.017	0.089

**Table 3:** The result of TDC analysis (month).

	T_month_	RH_month_	PM2.5_month_	PM10_month_	SO_2,month_	NO_2,month_
$r_{h}$	0.172	−0.201	0.025	0.020	−0.083	−0.040
$r_{v}$	–	–	–	–	–	–

**Table 4:** The result of maximal information coefficient (MIC).

	T	RH	PM2.5	PM10	SO_2_	NO_2_
Part 1	0.979	0.724	0.506	0.509	0.488	0.464
Part 2	0.999	0.983	0.696	0.761	0.717	0.605
Part 3	0.947	0.524	0.404	0.379	0.347	0.379
